# Aerobic exercise, an effective prevention and treatment for mild cognitive impairment

**DOI:** 10.3389/fnagi.2023.1194559

**Published:** 2023-08-08

**Authors:** Baiqing Huang, Kang Chen, Ying Li

**Affiliations:** ^1^Sports Institute, Yunnan Minzu University, Kunming, China; ^2^Tianjin Key Lab of Exercise Physiology and Sports Medicine, Tianjin University of Sport, Tianjin, China

**Keywords:** mild cognitive impairment, aerobic exercise, risk factor, BDNF, crosstalk

## Abstract

Aerobic exercise has emerged as a promising intervention for mild cognitive impairment (MCI), a precursor to dementia. The therapeutic benefits of aerobic exercise are multifaceted, encompassing both clinical and molecular domains. Clinically, aerobic exercise has been shown to mitigate hypertension and type 2 diabetes mellitus, conditions that significantly elevate the risk of MCI. Moreover, it stimulates the release of nitric oxide, enhancing arterial elasticity and reducing blood pressure. At a molecular level, it is hypothesized that aerobic exercise modulates the activation of microglia and astrocytes, cells crucial to brain inflammation and neurogenesis, respectively. It has also been suggested that aerobic exercise promotes the release of exercise factors such as irisin, cathepsin B, CLU, and GPLD1, which could enhance synaptic plasticity and neuroprotection. Consequently, regular aerobic exercise could potentially prevent or reduce the likelihood of MCI development in elderly individuals. These molecular mechanisms, however, are hypotheses that require further validation. The mechanisms of action are intricate, and further research is needed to elucidate the precise molecular underpinnings and to develop targeted therapeutics for MCI.

## 1. Introduction

The World Health Organization reported in 2021 that over 55 million people globally suffer from dementia, a figure that is projected to reach 78 million by 2030 and 139 million by 2050. MCI, defined as a stage intermediate between normal cognition and dementia, is prevalent in the elderly population, and its frequency increases with age. However, despite MCI’s common occurrence, awareness about this condition remains low, and many patients delay medical consultation. Consequently, MCI patients are more prone to developing Alzheimer’s disease (AD) (Hanseeuw et al., 2019). The growing number of MCI sufferers presents a substantial health and economic burden globally, underscoring the urgency for effective disease management strategies. In the field of clinical research, physical exercise as a non-invasive intervention has gained extensive research attention for dementia prevention and intervention (Hillman et al., 2014) (Table 1). Clinical studies have indicated that aerobic exercise outperforms anaerobic exercise in improving cognition in middle-aged and elderly adults (Liu-Ambrose et al., 2010). The therapeutic efficacy of aerobic exercise on MCI has been highlighted in various studies (Karssemeijer et al., 2017). For instance, Frisoni et al. (2023) reported overall cognitive improvement in mild to moderate cognitive impairment following a 6-month aerobic exercise intervention. Similarly, Tsai et al.’s (2019) 16-week aerobic exercise intervention study on MCI patients confirmed these findings (Yu et al., 2021). In the experimental research context, the role and mechanism of aerobic exercise in improving cognition have become a popular research topic. Recent experimental studies, however, have revealed that aerobic exercise may not effectively intervene or prevent AD, but intriguingly, it appears more beneficial for preventing and improving pre-AD MCI (Prince et al., 2013). The World Health Organization (WHO) endorses sustained aerobic exercise for individuals with dementia or at high risk of dementia. This lifestyle intervention has been shown to effectively manage or prevent disease progression (Rao et al., 2018). Additionally, a study by Giovanni et al. identified aerobic exercise as a form of cognitive training (Rao et al., 2018).

**TABLE 1:** Definition of different forms of exercise.

Term	Definition	References
Exercise	A subcategory of physical activity that is planned, structured, repetitive, and purposeful in the sense that the improvement or maintenance of one or more components of physical fitness is the objective.	WHO, 2020
Aerobic exercise	Activity in which the body’s large muscles move in a rhythmic manner for a sustained period, where oxygen demand does not surpass oxygen supply. Examples include bicycling, walking, swimming, and running.	WHO, 2020
Anaerobic exercise	Anaerobic physical exercise consists of brief intense bursts of exercise, such as weightlifting, sprinting, and High-intensity interval training, where oxygen demand surpasses oxygen supply.	WHO, 2020
Physical activity	Any bodily movement produced by skeletal muscles that requires energy expenditure.	WHO, 2020

## 2. Distress for MCI patients

### 2.1. Risk factors for MCI

**FIGURE 1:**
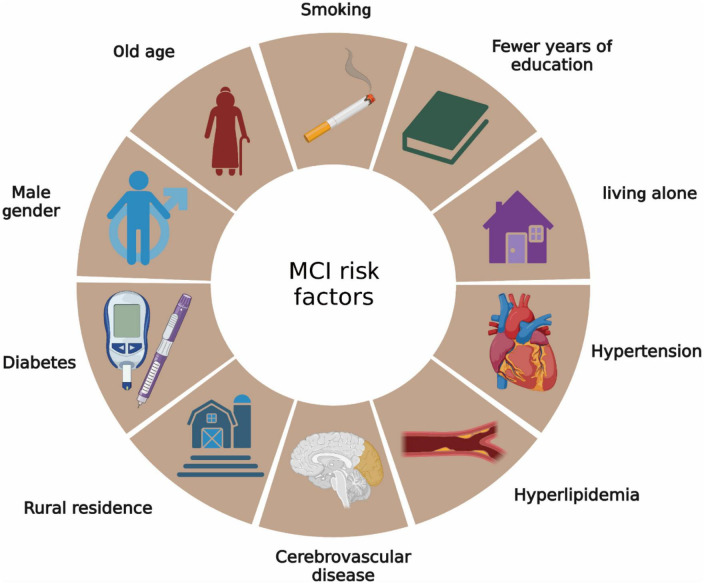
Risk factors for MCI. Risk factors for MCI are very similar to Alzheimer’s disease.

Mild cognitive impairment and dementia share several common risk factors, such as old age, female sex, a parental history of dementia, rural residence, limited education years, living situations such as being widowed, divorced, or living alone, and lifestyle and health conditions including smoking, hypertension, hyperlipidemia, diabetes, heart disease, and cerebrovascular disease (Jia et al., 2020) (Figure 1). Of these, nine are modifiable. Aerobic exercise has been recognized as a preventive measure that could potentially reduce two of these risk factors, specifically hypertension and diabetes (Jia et al., 2020). In the clinical context, hypertension is a prevalent condition in elderly individuals, affecting approximately two-thirds of those over the age of 60 (Ungvari et al., 2021). It significantly increases the risks of vascular cognitive impairment and MCI. Diabetes also emerges as another substantial risk factor for MCI. Elderly individuals with diabetes are more susceptible to age-related cognitive decline than non-diabetic individuals. Spauwen et al. (2013) demonstrated that subjects with diabetes experienced more pronounced cognitive decline over 12 years, especially in aspects such as information processing speed and word recall capacity. Similarly, Zilliox et al. (2016) identified common molecular and cellular characteristics in symptoms of diabetes mellitus type 1 (T1DM), diabetes mellitus type 2 (T2DM), insulin resistance, memory deficits, and cognitive decline in older people. Experimentally, hypertension and diabetes impact cognitive function through several molecular and cellular mechanisms. Hypertension can trigger cognitive dysfunction by impairing the structure and functional integrity of the cerebral microcirculation, inducing oxidative stress, and affecting the blood-brain barrier (BBB), which in turn promotes neuroinflammation and the production of amyloid-beta (Aβ) protein in the brain (Nagai et al., 2010). Additionally, hypertension also contributes to the formation of cerebral atherosclerotic plaques, potentially leading to cerebral ischemic stroke and cognitive decline in elderly individuals. On the other hand, chronic hyperglycemia in diabetes can induce hippocampal dysfunction, leading to cognitive decline. For instance, Wang et al. (2017) disclosed that elevated levels of histone deacetylase IIa in the brains of diabetic subjects contribute to cognitive decline in older and obese individuals. The specific mechanism involves insulin resistance leading to a decrease in Akt activation, a protein significant in glucose metabolism and GSK3β inhibition. This induces the formation of cerebral tau tangles, thereby diminishing the cognitive ability of diabetic patients. Additionally, diabetes can activate the NF-kB pathway in the hypothalamus, leading to the release of numerous inflammatory factors that harm neural progenitor cells (NPCs) (Lang et al., 2009).

### 2.2. Glial cell activation and inflammatory response

Recent studies have revealed a significant role of microglia and astrocytes in the development of MCI. Microglia, the innate immune cells of the central nervous system (CNS), are crucial for maintaining CNS homeostasis and performing a range of physiological functions, including regulation of neurogenesis, synaptic pruning, and production of trophic factors (Nayak et al., 2014; Prinz et al., 2019; Borst et al., 2021). They exist in three functional states: M0, M1, and M2 (Colonna and Butovsky, 2017). The M0 state is associated with nervous system development and reflects the functions of adult microglia crucial for maintaining homeostasis. The M1 state exhibits high expression of inflammation-related transcripts, producing cytokines such as tumor necrosis factor (TNF), interleukin-1β (IL-1β), nitric oxide (NO), and reactive oxygen species (ROS). The M2 state expresses genes that promote tissue development and neural cell renewal and enhance the production of anti-inflammatory cytokines such as IL-4 and IL10 (Colonna and Butovsky, 2017). In MCI, it has been observed that brain microglia adopt an activated M1 morphology, which stimulates the production of proinflammatory cytokines and promotes the production of Aβ proteins. Thus, microglia-mediated inflammation is a key component of the immune response in MCI, and addressing this response is critical for mitigating chronic inflammation in the MCI brain (Gamage et al., 2020).

Astrocytes, the most abundant, diverse, and functionally versatile cell type in the brain, also play a significant role (Freeman, 2010). They are involved in multiple functions, including supporting and populating the brain parenchyma, isolating, and insulating damaged tissue, participating in BBB formation, synaptic plasticity, ion transfer, and energy metabolism, and synthesizing and metabolizing neurotransmitters and neurohormones. Astrocytes exist in two functional states, A1 and A2. The A1 state induces inflammation and loses its capacity to protect neurons and regulate synaptogenesis, transforming into phagocytic cells that trigger neuronal loss through the NF-kB pathway (Liddelow et al., 2017). Conversely, the A2 state enhances the production of neurotrophic factors, promoting neuronal survival via the Janus kinase/signal transducer and activator of transcription 3 (JAK-STAT3) signaling pathway (Wang et al., 2020), which produces neurotrophic factors such as brain-derived neurotrophic factor (BDNF) (Gamage et al., 2020). In MCI, astrocytes are among the first cells to be activated during disease progression (Garcia et al., 2022). Notably, abnormally activated astrocytes express BACE1 and increase Aβ production while also reducing the degradation and clearance of Aβ, leading to its accumulation in the brain (Guillamon-Vivancos et al., 2015). Concurrently, activated M1 microglia can induce astrocytes to transition into proinflammatory, cytotoxic A1 astrocytes, intensifying the inflammatory response in the MCI brain. Furthermore, astrocytes facilitate synaptic formation during glycolysis through glutamate metabolism and other coupling mechanisms. In MCI, molecules related to these processes are disrupted, leading to glucose metabolism disorders. Thus, in MCI, microglia and astrocytes transition into a proinflammatory state, ultimately leading to Aβ accumulation and metabolic disorders in the brain.

## 3. Benefits of aerobic exercise for MCI

Clinical outcomes indicate that aerobic exercise has been widely recognized for its potential to mitigate global cognitive decline and behavioral issues in individuals with MCI. Notably, aerobic exercise of moderate intensity or above has a more significant effect on cognitive performance in individuals with MCI (Law et al., 2020). Several recent retrospective studies have further substantiated the positive effect of aerobic exercise on cognitive performance in patients with MCI (Karssemeijer et al., 2017; Petersen et al., 2018; Demurtas et al., 2020). Importantly, aerobic exercise can reduce the risk factors associated with MCI. Of the numerous risk factors for MCI, nine are modifiable, and aerobic exercise emerges as a promising intervention for two of these: hypertension and diabetes. In clinical settings, physicians often perceive aerobic exercise as an effective supplementary treatment for both hypertension and diabetes (Saco-Ledo et al., 2020; Sakamoto, 2020).

Moving on to the experimental domain, the results show that aerobic exercise improves the activation of glial cells and the inflammatory response in the MCI brain. It has been established that even low-intensity aerobic exercise is sufficient to counteract the effects produced by the activation of microglia by modulating the expression of various factors. Among these, aerobic exercise generates myokines that directly inhibit the activation of microglia through various mechanisms, thereby preventing neuroinflammation in the central nervous system. An extensive body of evidence also suggests that aerobic exercise may inhibit microglial activation by downregulating proinflammatory factors (Mee-Inta et al., 2019). Moreover, several factors produced as a result of aerobic exercise have a positive physiological effect on MCI. These include Irisin, Cathepsin B, CLU (clusterin), and Glycosylphosphatidylinositol-specific Phospholipase D1 (GPLD1). These have all been widely reported to positively impact the brains of MCI patients through multiple pathways.

### 3.1. Aerobic exercise to prevent MCI by reducing risk factors for MCI

Numerous risk factors contribute to MCI, some of which are modifiable, and others that are not. Therefore, reducing the modifiable risk factors for MCI could be a viable preventive measure. Aerobic exercise has been identified as an effective strategy to reduce some of these risk factors, notably hypertension and diabetes (Liu et al., 2019). For the hypertensive population, aerobic exercise is a non-pharmacological intervention that has been widely reported to alleviate hypertension. In clinical practice, physicians often consider aerobic exercise as an important adjunct therapy for patients with hypertension. Research has shown that blood pressure (BP) can remain lower for up to 24 h following aerobic exercise, with the greatest reduction observed in individuals with the highest baseline BP (Alpsoy, 2020). Aerobic exercise can prevent BP increases in normal adults and lower BP in hypertensive individuals. Their BP can significantly drop by 5 to 7 mm Hg after aerobic exercise (Freeman, 2010). Exercise-induced reductions in BP are due to neurohumoral regulation and the adaptation of blood vessels and structures. Therefore, many professional organizations recommend moderate aerobic exercise for individuals with hypertension, at least 30 min per session, 3 days a week.

Recently, the mechanisms by which exercise alleviates hypertension have been further explored. After aerobic exercise, nitric oxide (NO) levels in the body increase, which improves arterial stiffness and reduces vascular resistance and peripheral arterial vascular tension, consequently lowering BP (Law et al., 2020). Vanhoutte et al. (2017) found that the beneficial effects of exercise training on BP were associated with an improvement in NO/cGMP-mediated vascular relaxation. Additionally, aerobic exercise has been confirmed to be highly beneficial for people with type 2 diabetes mellitus (T2DM). It enhances metabolic control and insulin sensitivity and reduces inflammatory markers and neuropathic symptoms. Aerobic exercise could impact type 1 diabetes mellitus (T1DM) through immune modulation (He et al., 2017). Regular aerobic exercise has shown benefits to body composition, cardiovascular integrity, and insulin sensitivity (de Senna et al., 2015). Furthermore, it has been proven that aerobic exercise can effectively improve waist and hip circumferences, plasma insulin, and insulin resistance in T2DM patients. Other studies found that both aerobic and resistance exercises could effectively control T2DM (Chupel et al., 2018). Moreover, the mechanisms by which aerobic exercise improves diabetes have been further investigated. Aerobic exercise increases the number of glucose transporters (GLUTs) on muscle cell and adipocyte membranes, facilitating the transport and utilization of glucose (Pang et al., 2019). It strengthens insulin receptor function, improving the sensitivity of peripheral tissues to insulin, thereby reducing insulin resistance. Therefore, aerobic exercise can improve diabetes, reduce the risk factors for MCI, and further ameliorate or delay MCI.

**FIGURE 2:**
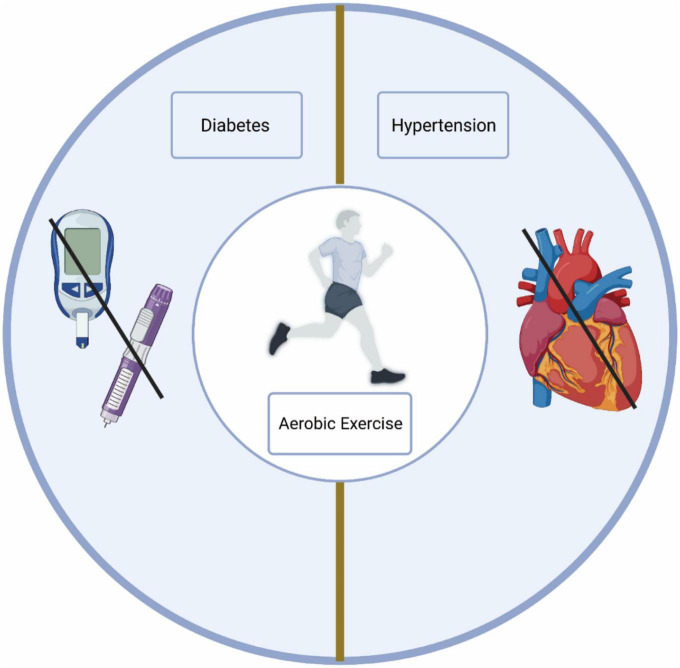
Aerobic exercise reduces the risk of MCI. Aerobic exercise reduces the risk of diabetes and hypertension.

In conclusion, aerobic exercise has proven to be an effective intervention for hypertension and diabetes, reducing the risk of MCI in older adults. Regular aerobic exercise for elderly individuals could potentially prevent or reduce the likelihood of developing MCI (Figure 2).

### 3.2. Molecular underpinnings of aerobic exercise in the treatment and prevention of MCI

#### 3.2.1. Aerobic exercise reduces glial cell activation and the inflammatory response in MCI

Glial cell activation and the inflammatory response are central to the progression of MCI, playing significant roles in the cognitive decline experienced by MCI patients (He et al., 2017). In recent times, the effects of these factors on MCI, as well as potential strategies for mitigating MCI progression through the reduction of glial cell activation and inflammation, have emerged as a keen area of focus. Notably, researchers have identified aerobic exercise as a crucial element in alleviating both glial cell activation and the inflammatory response. Consequently, these findings significantly enhance our understanding of the role and mechanism of aerobic exercise in MCI improvement.

Microglia and neurons share an intricate crosstalk relationship. Notably, microglial activation is largely associated with neuronal dysfunction and damage. However, aerobic exercise has the capacity to alleviate or inhibit such damage to neurons through microglia modulation (Mee-Inta et al., 2019). Aerobic exercise, by increasing anti-inflammatory factors, influences both microglial activation and neural regeneration. Primarily, aerobic exercise reduces inflammation by decreasing the release of inflammatory cytokines from skeletal muscle, a process that occurs independently of weight loss. Notably, skeletal muscle, the body’s largest organ, produces and releases inflammatory cytokines such as IL-4, IL-6, IL-8, IL-15, and TNF-α, akin to adipose tissue and immune cells (Mee-Inta et al., 2019). In contrast, Handschin and Spiegelman (2008) discovered that aerobic exercise broadly inhibited myokine expression through the upregulation of skeletal muscle peroxisome proliferation-activated receptor 1α. Postexercise, there have been observations of increased circulating levels of IL-6 without muscle damage. IL-6, by stimulating the anti-inflammatory cytokines IL-1ra and IL-10, reduces the release of pro-inflammatory cytokines, ultimately leading to a decrease in overall inflammation. Moreover, aerobic exercise confers a protective effect on the structure of the BBB. de Senna et al. (2015) reported that aerobic exercise improved the structural composition of the BBB in diabetic rats. This phenomenon was replicated in human studies where recent research demonstrated that aerobic exercise helped maintain BBB integrity. These studies examined the anti-inflammatory effects of combined exercise training and taurine supplementation on peripheral markers of BBB integrity, inflammation, and cognition in 48 elderly women (Chupel et al., 2018). The results indicated that the combined exercise training group experienced reductions in TNF-α and IL-6, alongside diminished IL-1β/IL-1ra, IL-6/IL-10, and TNF-α/IL-10 ratios. Furthermore, aerobic exercise regulates microglial activation by upregulating CD200-CD200R, TREM2, and heat-shock protein (HSP) levels. In essence, aerobic exercise can regulate microglial activation via multiple pathways. In parallel, aerobic exercise lessens the level of inflammation in the patient’s brain by safeguarding the structural integrity of the BBB.

**FIGURE 3:**
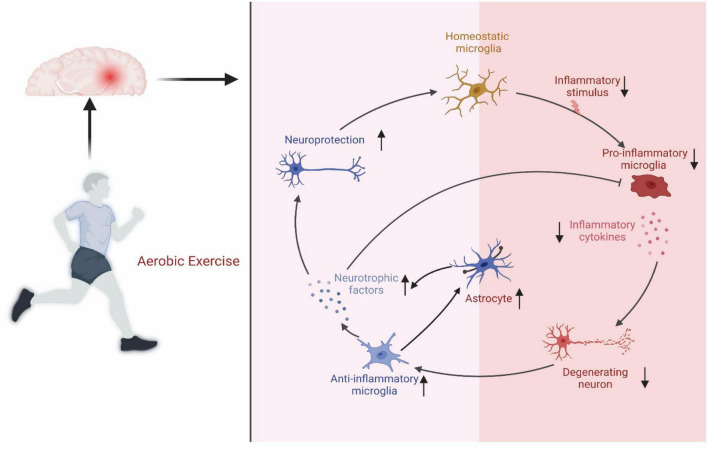
Regulatory mechanisms on aerobic exercise on microglia and astrocytes. Aerobic exercise improves microglia and astrocytes status to protect nerves.

For astrocytes, aerobic exercise stimulates their proliferation. It lays the cellular basis for aerobic exercise, mediating neurogenesis and improving cognitive function (He et al., 2017). Aerobic exercise activates the expression of astrocytes by transcribing different mRNAs, ultimately leading to structural and functional changes in astrocytes. In turn, astrocytes can promote the growth and differentiation of neuronal cells by releasing various factors, thus improving the cognitive performance of MCI patients (Zhou et al., 2019). Glutamate is an essential excitatory neurotransmitter that stimulates presynaptic N-methyl-D-aspartate receptors (NMDAR) through astrocyte activation (Li et al., 2021). Aerobic exercise improved neuroplasticity by increasing glutamate secretion by astrocytes and NMDAR expression in neurons. D-Serine, a neurotransmitter released by astrocytes, was found to play a role in NMDAR-dependent long-term potentiation (LTP) (Li et al., 2021). Moreover, aerobic exercise induces the release of D-serine from astrocytes. These mechanisms further explain why aerobic exercise improves the cognitive function of MCI patients through the interaction of astrocytes and neurons. In addition, astrocyte activation attenuates or delays BBB damage in MCI (Agudelo et al., 2014; He et al., 2017). In conclusion, the role of aerobic exercise in improving cognitive function in MCI patients by modulating microglia and astrocyte status and function cannot be ignored (Figure 3).

#### 3.2.2. Aerobic exercise improves cognitive performance in MCI by promoting exercise factor release

Aerobic exercise is achieved primarily through the contraction of skeletal muscles, so how do skeletal muscles talk to the brain? Currently, skeletal muscle is considered the body’s secretory organ, releasing various exercise factors into the circulatory system to crosstalk with other body organs. Aerobic exercise promotes the secretion of exercise factors and hormonal molecules from peripheral tissues and organs (including skeletal muscle) to act on neurons in the brain directly or after modulating downstream proteins across the BBB. On the one hand, the entry of exercise factors into the brain promotes the secretion of neurotrophic factor (BDNF), increases synaptic plasticity, and has protective effects on neurons (Chen et al., 2022). On the other hand, they improve the inflammatory environment and alleviate the inflammatory microenvironment in the brain (Townsend et al., 2021). These factors mainly include irisin, cathepsin B (CTSB), CLU, and glycosylphosphatidylinositol-specific phospholipase D1 (GPLD1) (Lu et al., 2023). These exercise factors can protect the normal structure of the brain and enhance cognitive function through all the processes mentioned above.

Irisin is considered a motility inducer regulated by peroxisome proliferator-activated receptor gamma coactivator 1-alpha (PGC1α), which acts via the circulatory system in various parts of the body after shearing from precursor FNDC5 (Kim et al., 2018). For example, irisin induces white fat browning and thermogenesis (Chen et al., 2022). Interestingly, irisin can positively affect cognitive function in the brain through multiple pathways. Aerobic exercise promoted PGC1-α expression via the AMPK and p38MAPK pathways, leading to increased irisin expression and, ultimately, positive effects on cognitive function. A study by Chen et al. (2022) found that aerobic exercise increased irisin expression in the hippocampus and positively affected cognitive function. To demonstrate the relationship between irisin and cognitive impairment, Lourenco et al. (2019) injected FNDC shRNA into the mouse brain, which impaired long-term hippocampal enhancement, markers of synaptic plasticity and cognitive function. Similarly, increasing mouse brain irisin content by injection of recombinant adenovirus improved cognitive dysfunction and synaptic plasticity in mice. A large body of evidence points to a neuroprotective and cognitive improvement effect of irisin, and whether irisin acts after aerobic exercise deserves further investigation. A study by Lourenco et al. (2019) found that aerobic exercise improved brain Aβ protein deposition and cognitive function. To determine whether this is an effect of peripheral irisin or local irisin, they further demonstrated that aerobic exercise improves cognitive function and synaptic plasticity by promoting irisin expression using tail vein adenoviral injections of irisin combined with aerobic exercise intervention in mice. The molecular mechanisms by which irisin improves cognitive function are gradually being revealed. Irisin has also been shown to improve cognitive function after aerobic exercise. It crosses the BBB through the circulatory system into the central nervous system. It activates the cAMP/PKA/CREB and MAPK/ERK pathways, increasing BDNF in neurons (Chen et al., 2022; Lu et al., 2023). Recent studies have found that the cAMP/PKA/CREB pathway can reduce the burden of neural tangles in the brain by inhibiting the transcription and expression of tau proteins (Lu et al., 2023). In addition, inhibition of this pathway may also modulate brain neuroplasticity and cognitive dysfunction. A study by Lee et al. (2021) found that mice’s short-term memory improved after aerobic exercise. Activation of the MAPK/ERK pathway increased the expression of several cognitively relevant genes, such as Arc, Zwint, Arl5b, and Homer1 (Lu et al., 2023).

Cathepsin B is a cysteine protease widely expressed in humans and other mammals, mainly in lysosomes, but is also present in the cytoplasm, extracellular space, and some organelles (Xie et al., 2023). It plays a key role in protein degradation, apoptosis, the inflammatory response, autophagy, and cell migration (Chevriaux et al., 2020; Wang et al., 2020). For its protein degradation aspects, cathepsin B has been shown to reduce the levels of Aβ (Hook et al., 2022), a protein that reduces cognitive performance in MCI patients and may convert MCI to AD. Therefore, cathepsin B may have neuroprotective effects (Pedersen, 2019). Interestingly, aerobic exercise, but not resistance exercise, increases circulating muscle and hippocampal CTSB levels (Gaitan et al., 2021). Moon et al. (2016) found that aerobic exercise increased CTSB levels and mRNA expression in skeletal muscle. Importantly, their study also found that 4 months of aerobic exercise increased circulating levels of CTSB in rhesus monkeys and humans. In addition, CTSB can cross the BBB (Moon et al., 2016). Exercise-induced CTSB can circulate through the periphery into the brain and promote brain BDNF expression to improve cognition. Interestingly, the level of CTSB *in vivo* after aerobic exercise is directly proportional to cognitive performance (Lu et al., 2023). In summary, CTSB can be a muscle factor produced by aerobic exercise, and its circulating levels are proportional to cognitive performance.

Clusterin, also known as apolipoprotein J, is a highly conserved glycoprotein (Wu et al., 2012). It is widely expressed in several tissues, including the liver, brain, central nervous system, and cardiovascular system. In addition, LRP8 is a receptor for CLU, and its expression is highest in the brain (Wu et al., 2012). Therefore, it can be inferred that elevated CLU in the blood may bind to and benefit the LRP8 receptor in the brain. Interestingly, a study by De Miguel et al. (2021) found that aerobic exercise promoted CLU expression in mice, reduced inflammation and improved cognitive function in the mouse brain. They also conducted a 6-month aerobic exercise intervention in MCI patients and found higher plasma levels of CLU in the patients. To further explore the crosstalk between CLU and cognitive function, they injected exercise plasma without CLU in mice and found significantly lower levels of CLU and anti-inflammatory factors (De Miguel et al., 2021). Thus, aerobic exercise may modulate neuroinflammatory conditions and improve cognition by elevating CLU levels. CLU could remove Aβ protein aggregation and tau protein tangles, in addition to fighting neuroinflammation (De Miguel et al., 2021). However, specific mechanistic studies are scarce and need to be further explored. In summary, CLU may be one of the important mediators of aerobic exercise to improve cognition in the brain.

Glycosylphosphatidylinositol-specific Phospholipase D1 is a phospholipase that cleaves proteins anchored to the cell membrane by glycosylphosphatidylinositol (GPI) (Horowitz et al., 2020). Upon their release from the cell surface, these cleaved proteins perform different biological functions. Previous studies have emphasized the importance of GPLD1 in conditions such as diabetes and cancer. A recent study by Horowitz et al. (2020) broadened this scope, suggesting that GPLD1 also has a promising effect on cognition. They observed improved performance in the Trail-arm water maze (RAWM) and novel object recognition tasks in aging mice after a 6-week aerobic exercise intervention. This suggested not only an improvement in cognitive performance but also a concomitant increase in the hepatic secretion of GPLD1 expression (Horowitz et al., 2020). To determine if GPLD1 mediated the cognitive improvement associated with aerobic exercise, they implemented a technique of overexpressing GPLD1 in mice using tail vein injection of plasmids. Through behavioral and physiological assays, they concluded that GPLD1 positively influenced cognitive function. Notably, their study found that high levels of GPLD1 were only associated with the presence or absence of exercise and were not correlated with age or sex (Horowitz et al., 2020). Mechanistic studies suggest that GPLD1 does not directly cross the BBB but influences cognitive function primarily by affecting the expression of its downstream uPAR pathway-associated proteins (coagulation and complement system-associated proteins) (Horowitz et al., 2020). This led them to propose that GPLD1 is a key protein acting on the muscle-liver-brain axis. However, despite these advancements, there are still few studies on how exercise regulates GPLD1 expression and the mechanisms by which GPLD1 regulates cognitive performance.

**FIGURE 4:**
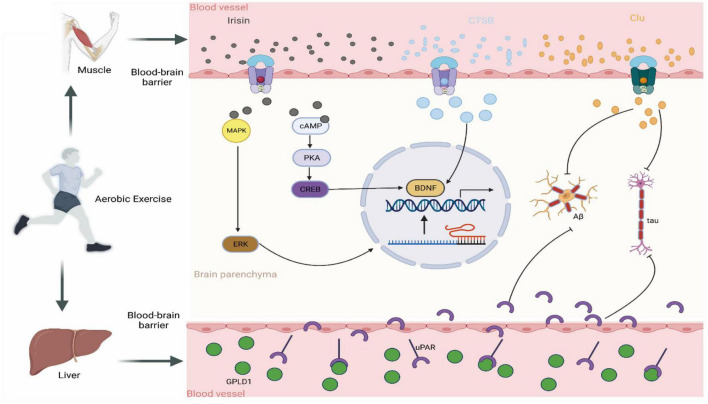
Crosstalk between brain and exercise-induced factors. Irisin: following aerobic exercise, skeletal muscle secretes the hormone irisin into the circulator system which subsequently penetrates the blood-brain barrier to gain access to the brain. Once there, Irisin stimulates BDNF production by activating the MAPK/ERK and cAMP/CREB pathways. CTSB: Post-aerobic exercise, skeletal muscle releases the protease CTSB into the circulatory system, which subsequently migrates to the brain and therein promotes BDNF production. CLU: Upon completion of aerobic exercise, skeletal muscle discharge-es the chaperone protein CLU into the circulatory system. This molecule subsequently traverses to the brain, where it binds to its receptor, thus ultimate-ly inhibiting Aβ aggregation and the formation of neurofibrillary tangles. GPLD1: Aerobic exercise promotes the expression of hepatic GPLD1, which, by hydrolyzing the downstream protein uPAR, exerts cognitive-improving effects.

In conclusion, aerobic exercise can act as a stimulus to induce the release of muscle- or liver-specific factors that positively affect cognitive function (Figure 4). This underscores the need for further research into the specific role of GPLD1 in this physiological process.

## 4. Conclusion

Several clinical studies have consistently confirmed the positive effects of aerobic exercise on cognitive function, particularly within the elderly population (Table 2). In the context of the general population, aerobic exercise reduces the risk of developing MCI, whereas for those already diagnosed with MCI, aerobic exercise appears to improve cognitive function. For instance, in one notable randomized trial consisting of 33 patients with amnestic mild cognitive impairment, researchers segregated the subjects into two groups–stretching and aerobic exercise groups–for intervention. The results suggested that aerobic exercise not only enhances cardiorespiratory fitness but also significantly improves concentration, executive capacity, and cognitive decline, especially in elderly females (Baker et al., 2010). Complementing this, in another study, a randomized survey, scholars introduced interventions in the form of aerobics and square dancing. Subsequent analysis of the results revealed that the cognitive ability of subjects in the aerobic exercise group was markedly higher than that in the control group. This was evident in the subjects’ performance in areas such as concentration, delayed recall, and language expression (Merom et al., 2016). Interestingly, an investigation led by Zhu et al. (2018) revealed that the cognitive level of elderly individuals with mild cognitive impairment could decline if they ceased exercising. This underscores the idea that the benefits of aerobic exercise are sustained by promoting the body’s physical function.

**TABLE 2:** Effects of aerobic exercise on MCI patients.

Subjects/Age (years)	Intervention groups	Exercise protocol	Main effects	References
120 mild cognitive impairment participants (over 60 years)	Control group: education control group Intervention group: aerobic exercise group	16-week aerobic stepping exercise training, 3 days/week, 60 min/day	Improved cognitive function; improved sleep quality	Song and Yu, 2019
69 mild cognitive impairment participants (over 60 years)	Control group: education control group Intervention group: Baduanjin group	24-week of Baduanjin training, 3 days/week, 60 min/day	Improved cognitive function; enhanced regional fluctuation and gray matter volume in the hippocampus and anterior cingulate cortex	Tao et al., 2019
100 mild cognitive impairment participants (mean age, 75 years)	Control group: education control group Intervention group: multicomponent aerobic exercise group	6-month multicomponent aerobic exercise training, 2 days/week, 90 min/day	Improved cognitive function; reduced whole brain cortical atrophy	Suzuki et al., 2013
60 mild cognitive impairment participants (between 50 and 85 years)	Control group: education control group Intervention group: aerobic dance group	3-month aerobic dance training, 3 days/week, 35 min/day	Improved cognitive function	Zhu et al., 2018
60 mild cognitive impairment participants (mean age, 66 years)	Control group: stretch training group Intervention group: aerobic exercise group	stretch training group: 12-month stretch training aerobic exercise group: 1, 10-week aerobic treadmill training, 3 days/week, 25–30 min/day 2, 10-week aerobic treadmill training, 3–4 days/week, 90 min/day 3, 7-month aerobic treadmill training, 4–5 days/week, 30–40 min/day	Improved cognitive function; increased cerebral blood flow	Thomas et al., 2020
152 mild cognitive impairment participants (mean age, 65 years)	Control group: education control group Intervention group: Tai Chi group	12-month aerobic exercise training, 131.4 min/week	Improved cognitive function; more increased regional activity of brain	Li et al., 2023

However, the mechanisms by which aerobic exercise modulates MCI are intricate and multifaceted, and molecular experiments are required for further investigation. Aerobic exercise is understood to modulate inflammatory responses in the brain (Li et al., 2021), which it achieves by improving the morphology and state of microglia and astrocytes, as well as reducing the burden of Aβ and tau proteins in the brain (He et al., 2017). Brain plasticity represents another target for aerobic exercise. For the elderly population, including both those diagnosed with MCI and those with normal cognitive function, the release of exercise factors through aerobic exercise can enhance brain plasticity. This enhancement is realized by bolstering the expression of brain-derived neurotrophic factors and synaptic plasticity. BDNF expression, in particular, stimulates the survival and regenerative repair of neurons across various brain regions (Ruiz-Gonzalez et al., 2021). Importantly, the structure and function of these neurons constitute the foundation of cognitive ability. Recent research has discovered that exercise-induced increases in hippocampal BDNF levels are thought to be vital for improving cognitive function (Erickson et al., 2011). Furthermore, aerobic exercise has been associated with increased hippocampal volume and the elevated expression of synaptic plasticity proteins, factors that both contribute to enhanced long-term potentiation (LTP). For instance, a study involving aerobic exercise training intervention for 66 MCI patients demonstrated a marked improvement in cognition, along with significant changes in neuroprotective growth factors. Aerobic exercise was found to notably increase the levels of BDNF and insulin-like growth factor (IGF-1) in MCI patients (Tsai et al., 2019; Feng et al., 2022). Additionally, it was observed to potentially raise the levels of serum vascular endothelial growth factor (SVEGF) (Tsai et al., 2019). Therefore, aerobic exercise is a good dementia prevention measure for older adults with normal cognitive function. In a similar vein, for people with MCI, aerobic exercise may delay the progression of MCI and improve cognitive dysfunction. However, the effect may vary with individual genetic characteristics, gender, MCI type, and exercise.

The pathway connecting aerobic exercise and cognitive function in the brain is diverse and intricate. Despite considerable advances in recent years, there is still much to uncover about the underlying molecular mechanisms. A comprehensive understanding of the interaction between aerobic exercise and cognitive function in MCI patients can significantly contribute to the prevention or delay of MCI onset in the general elderly population. It can also enhance cognitive function in MCI patients and provide a robust theoretical basis for the development of MCI-targeted therapeutics. However, it is crucial to remember that these remain hypotheses that require further study and validation.

## Author contributions

BH: writing, original draft preparation, conceptualization, and editing. KC: reviewing, validation, and editing. YL: supervision, methodology, reviewing, resources, and validation. All authors contributed to the article and approved the submitted version.
